# Supplementary material for: Nanostructured Cu2O Synthesized via Bipolar Electrochemistry
Source: Nanomaterials (Basel). 2019 Dec 15;9(12):1781. doi: 10.3390/nano9121781 (PMC6956072; doi:10.3390/nano9121781)
Supplement: Supplementary file 1 [file nanomaterials-09-01781-s001.pdf]

Supplementary Materials

Nanostructured Cu_2O Synthesized via Bipolar Electrochemistry

Steven McWilliams [1], Connor D. Flynn [1], Jennifer McWilliams [2], Donna C. Arnold [3], Ruri Agung Wahyuono [4], Andreas Undisz [5], Markus Rettenmayr [5] and Anna Ignaszak [1,*]

[1] Department of Chemistry, University of New Brunswick, Fredericton, NB E3B 5A3, Canada; Steven.McWilliams@unb.ca (S.M.); connor.flynn@unb.ca (C.D.F.)

[2] Department of Psychology, University of New Brunswick, Fredericton, NB E3B 5A3, Canada; Jennifer.Sanford@unb.ca

[3] School of Physical Sciences, University of Kent, Canterbury CT2 7NH, UK; d.c.arnold@kent.ac.uk

[4] Institute for Physical Chemistry and Abbe Center of Photonics, Friedrich-Schiller-Universität, 07743 Jena, Germany; ruri.wahyuono@uni-jena.de

[5] Otto Schott Institute of Materials Research, Chair of Metallic Materials, Friedrich-Schiller-Universität, 07743 Jena, Germany; Andreas.Undisz@uni-jena.de (A.U.); M.Rettenmayr@uni-jena.de (M.R.)

* Correspondence: Anna.Ignaszak@unb.ca; Tel.: +1-506-261-9128

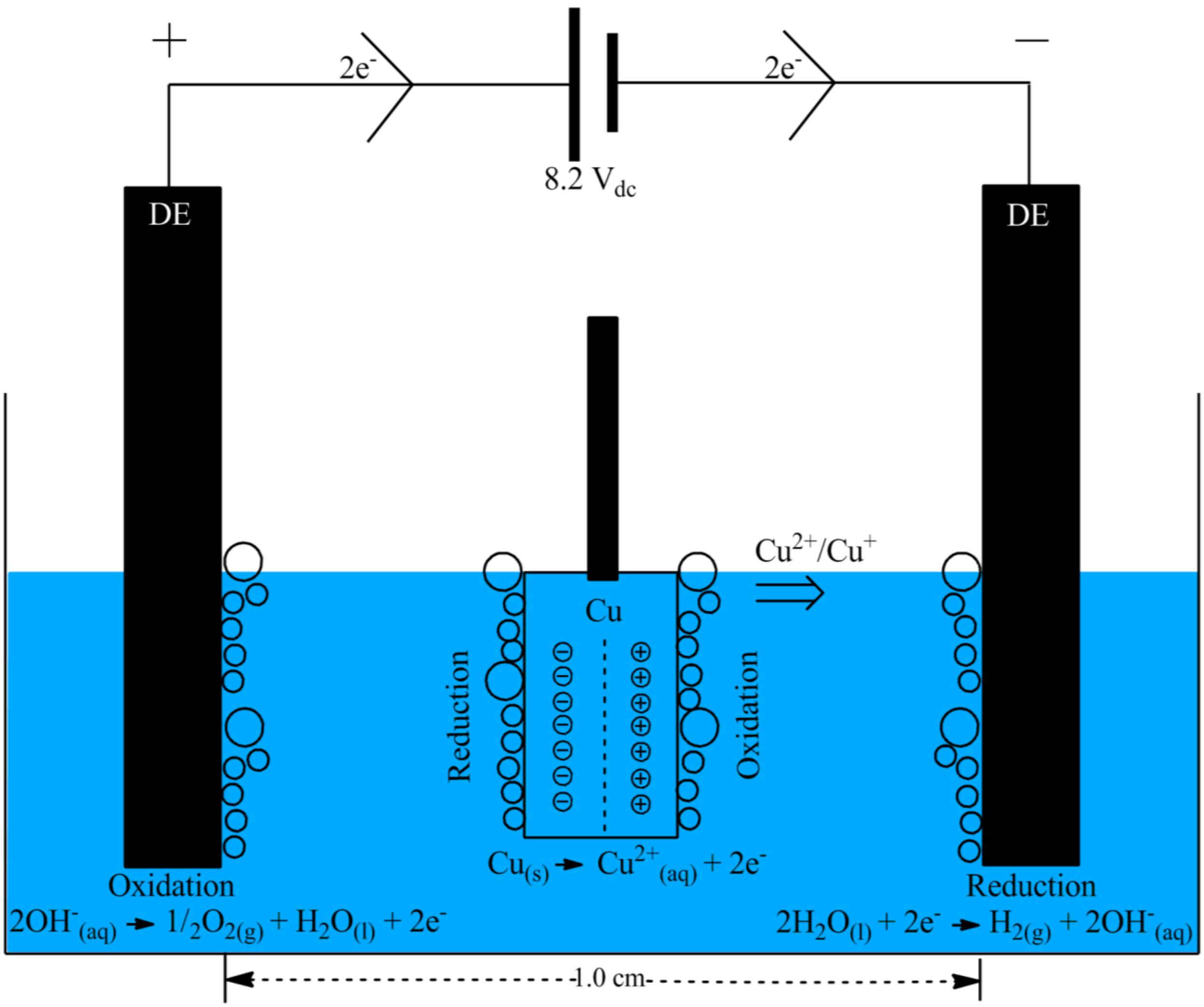

Figure S1: Schematic representing the bipolar electrochemical generation of copper ions for Cu_2O generation.

$Cu^0 \rightarrow Cu^{2+} + 2e^-$ Eq. S1.

$2H_2O + 2e^- \rightarrow H_{2(g)} + 2OH^-$ Eq. S2.

$Cu^{2+} + 2C_6H_{11}O_7{}^- \leftrightarrow C_{12}H_{22}CuO_{14}$ Eq. S3.

$2Cu^{2+} + C_6H_{12}O_6 + 5OH^- \rightarrow 2Cu_2O + C_6H_{11}O_7^- + 3H_2O$ Eq. S4.

$Cu^+ + OH^- \rightarrow CuOH$ Eq. S5.

$2CuOH \rightarrow Cu_2O + H_2O$ Eq. S6.

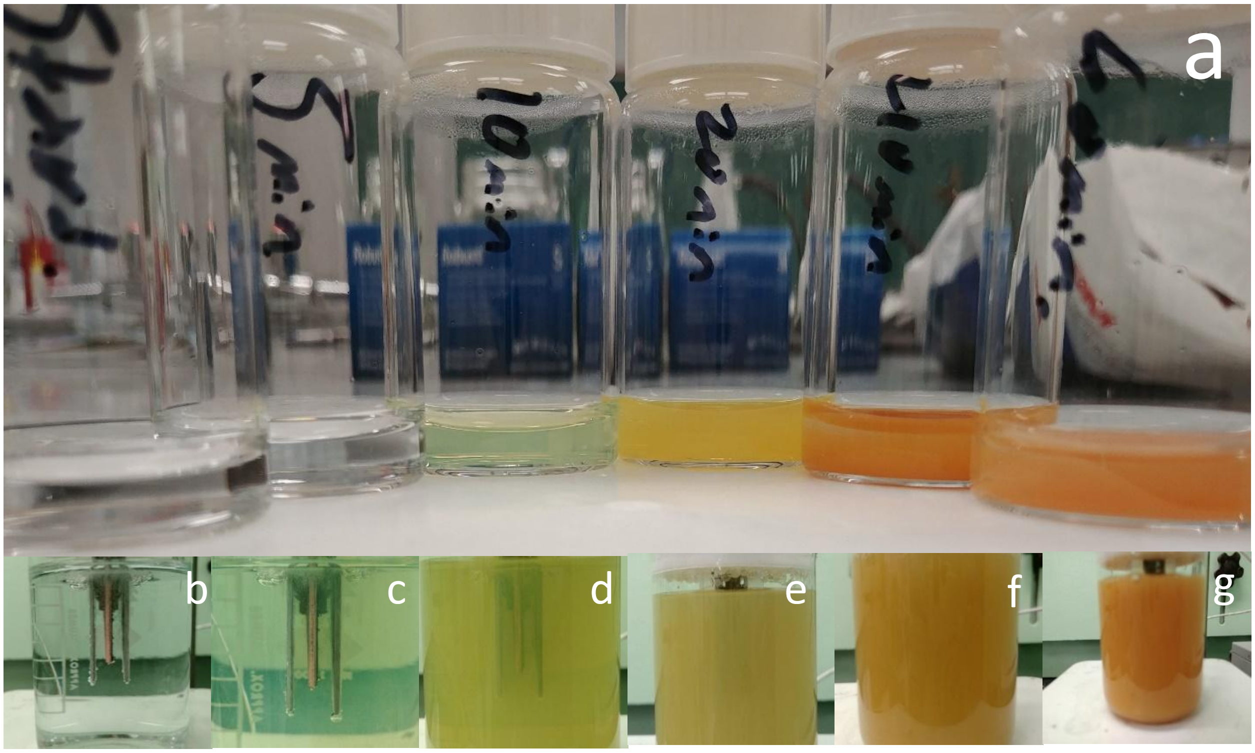

Figure S2: (a) Color change of the reaction solution during BPE synthesis over 60 min, from left: 0 min, 10, 20, 30, 40 and 60 min (right photo). (b-g) Photos taken during progression of the reaction inside the bipolar cell.

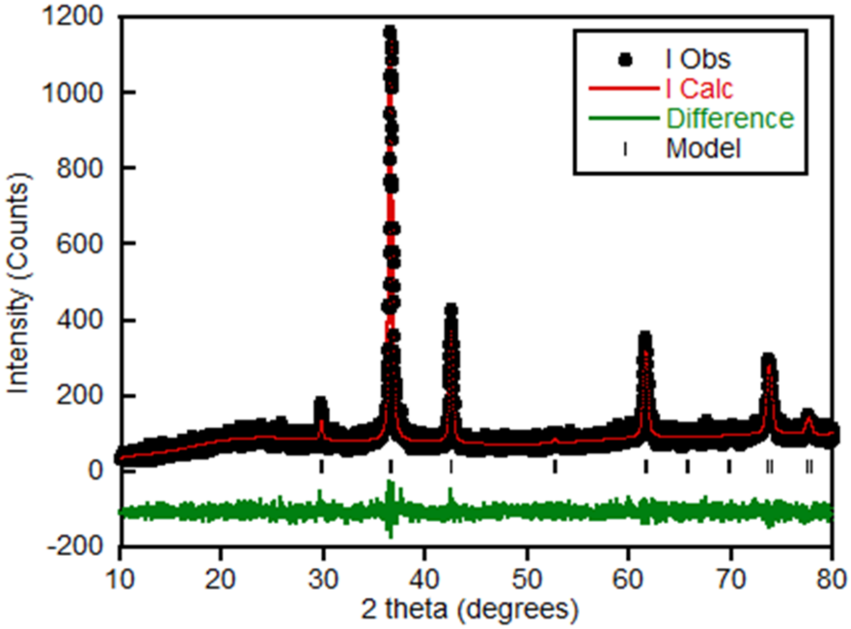

Figure S3: Refinement profile for the Rietveld analysis of room temperature XRD data that was collected for 4.5 V-3H and fitted to the model.[S1]

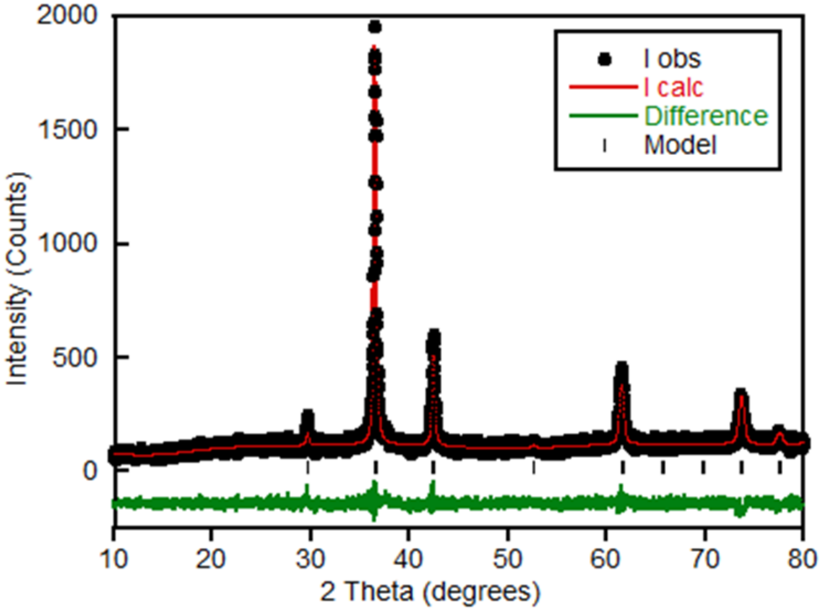

Figure S4: Refinement profile for the Rietveld analysis of room temperature XRD data that was collected for 4.5 V-1H and fitted to the model [S1].

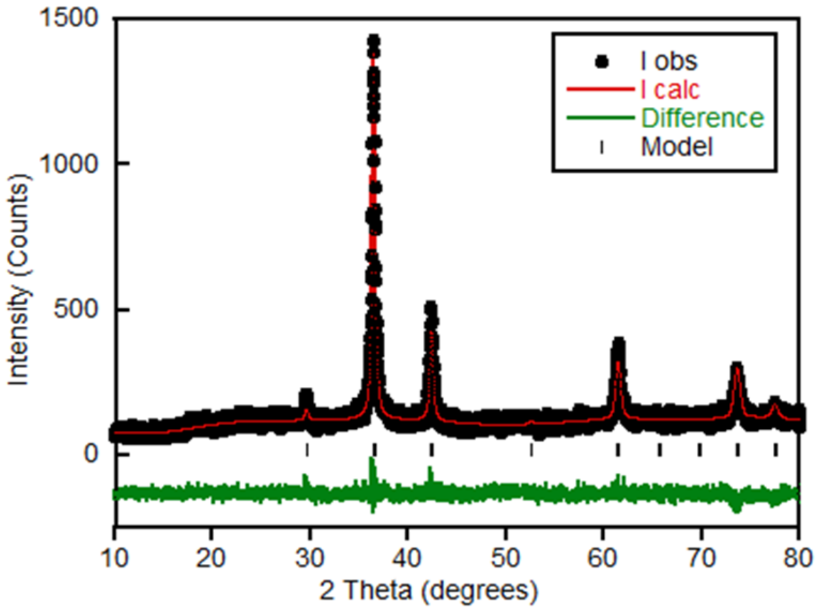

Figure S5: Refinement profile for the Rietveld analysis of room temperature XRD data that was collected for 5.0 V and fitted to the model.[S1]

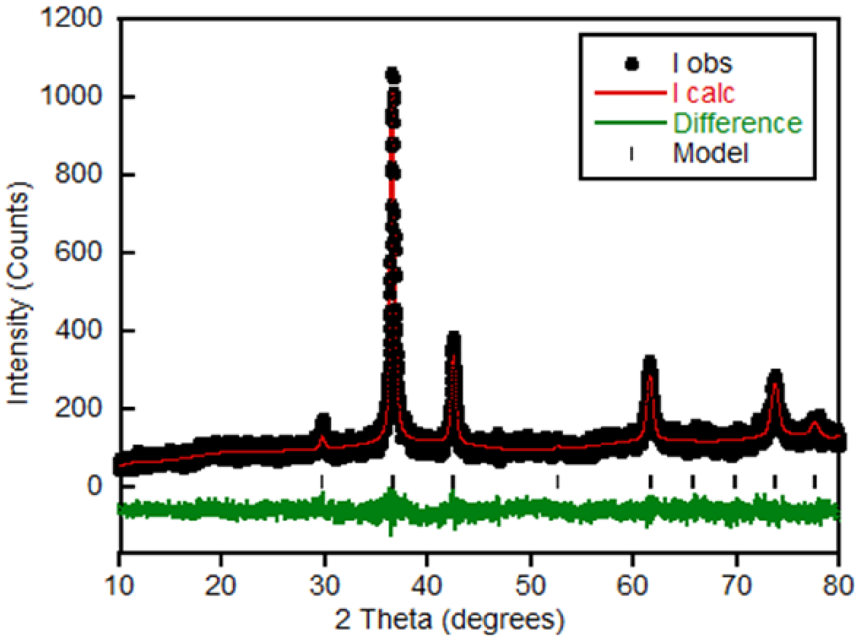

Figure S6: Refinement profile for the Rietveld analysis of room temperature XRD data that was collected for 6.0 V and fitted to the model.[S1]

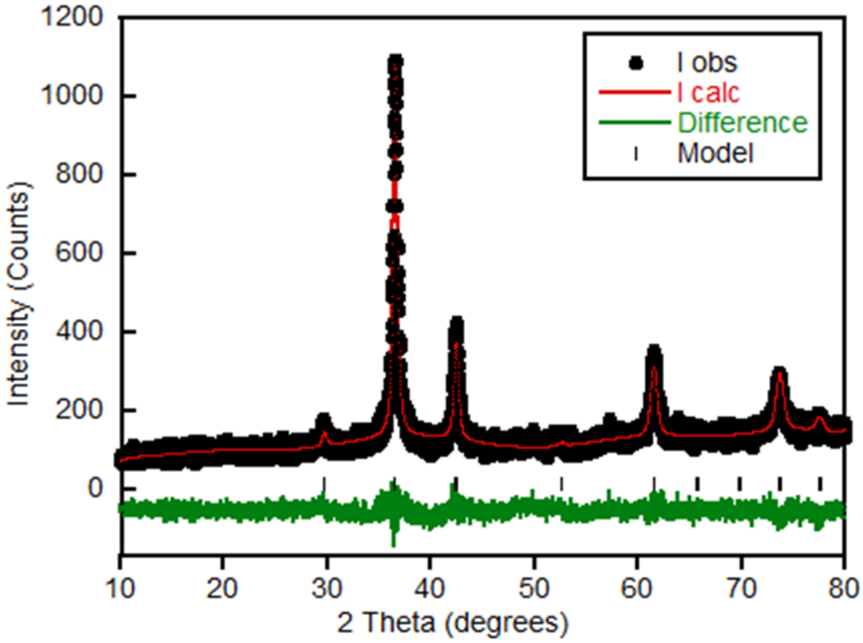

Figure S7: Refinement profile for the Rietveld analysis of room temperature XRD data that was collected for 7.0 V and fitted to the model.[S1]

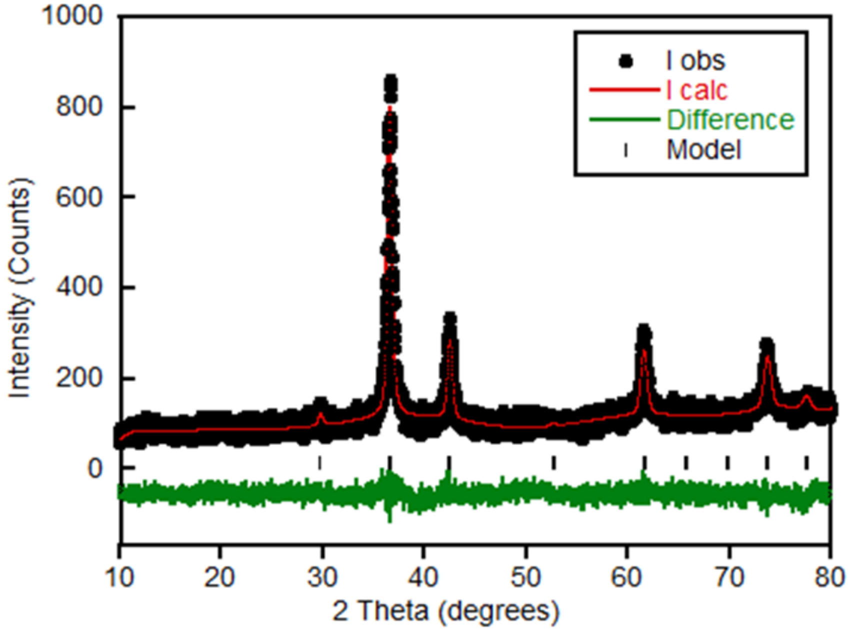

Figure S8: Refinement profile for the Rietveld analysis of room temperature XRD data that was collected for 8.0 V and fitted to the model.[S1]

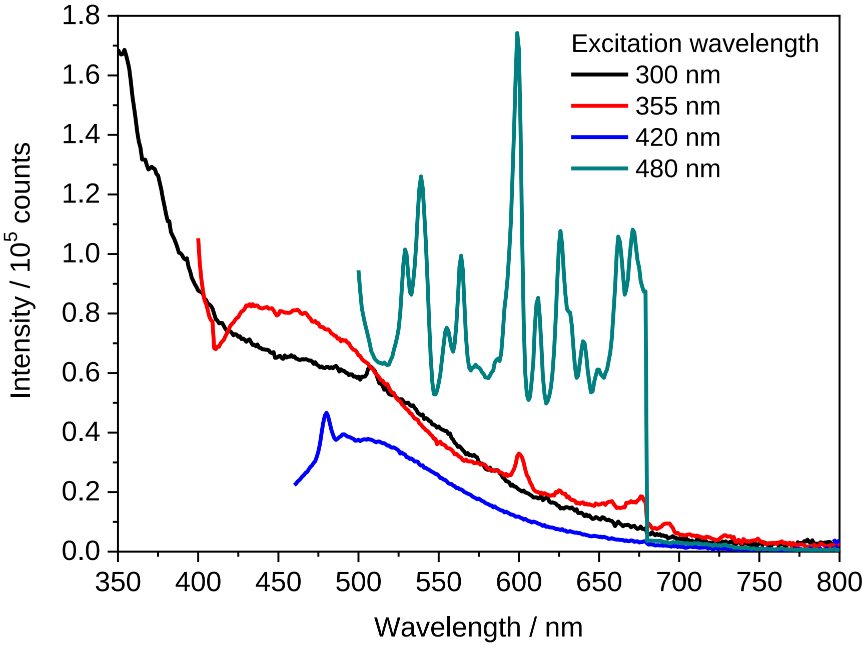

Figure S9: Emission spectra for Cu_2O synthesized by bipolar electrochemistry at 4.5 V (1h).

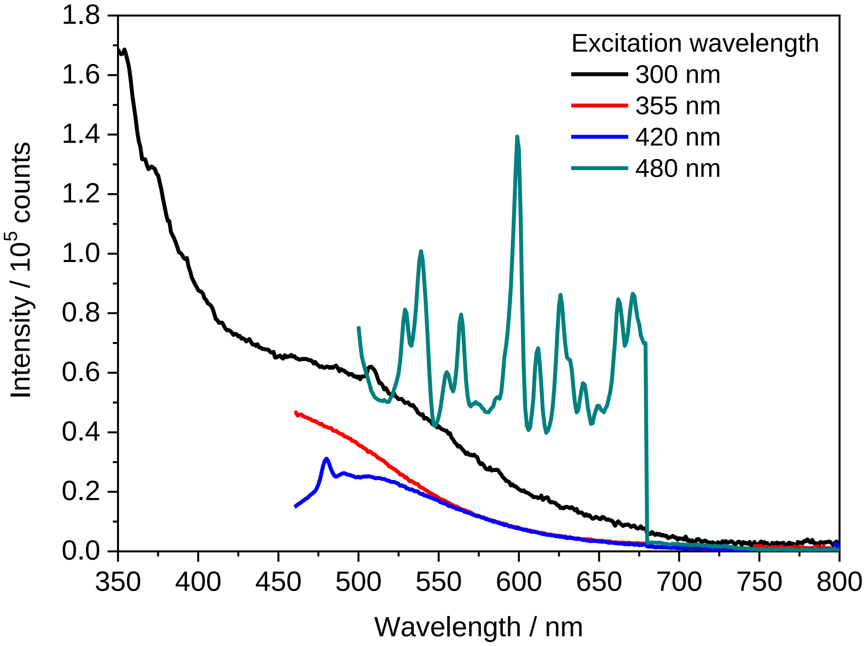

Figure S10: Emission spectra for Cu_2O synthesized by bipolar electrochemistry at 4.5 V (3h).

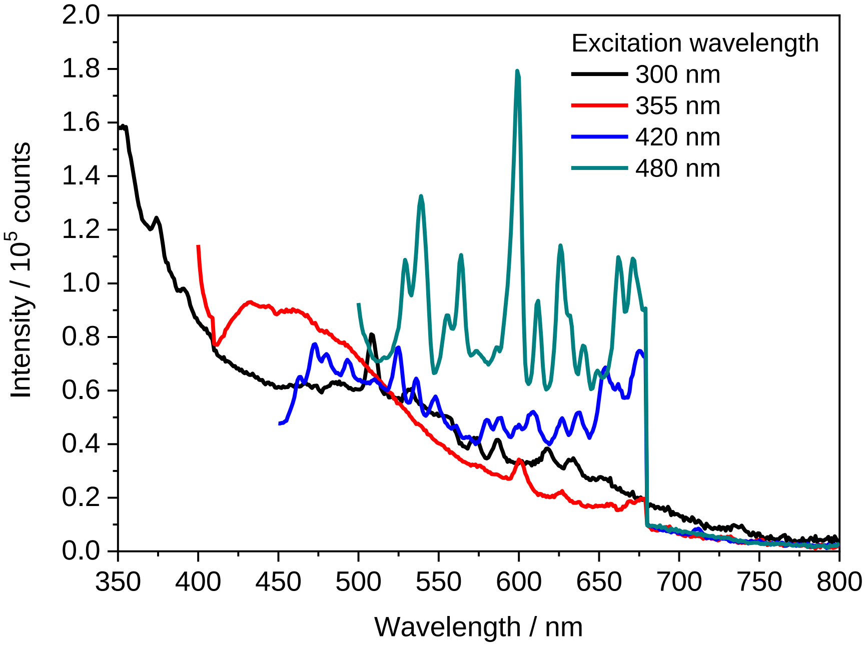

Figure S11: Emission spectra for Cu_2O synthesized by bipolar electrochemistry at 6.0 V (1h).

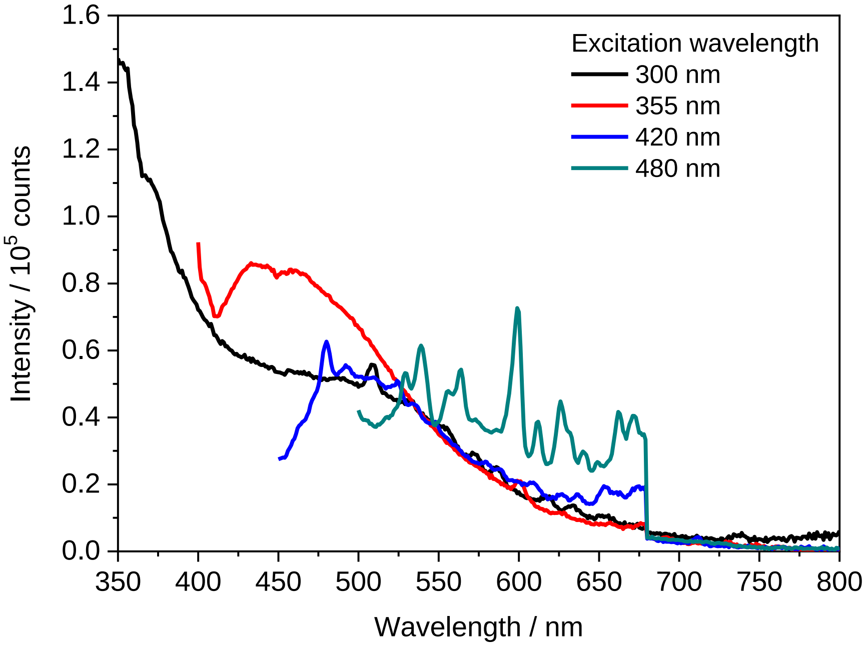

Figure S12: Emission spectra for Cu_2O synthesized by bipolar electrochemistry at 8.0 V (1h).

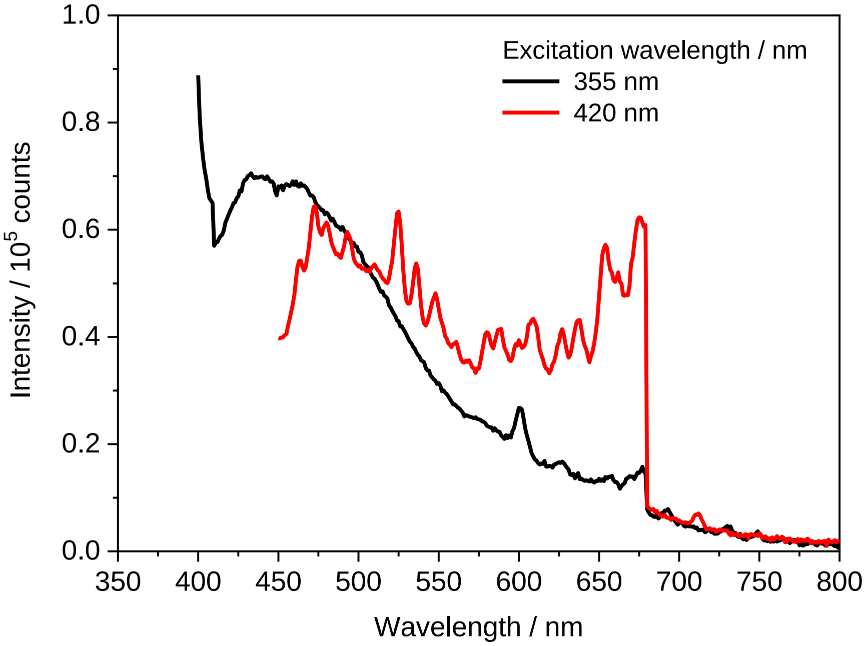

Figure S13: Emission spectra for commercial Cu_2O.

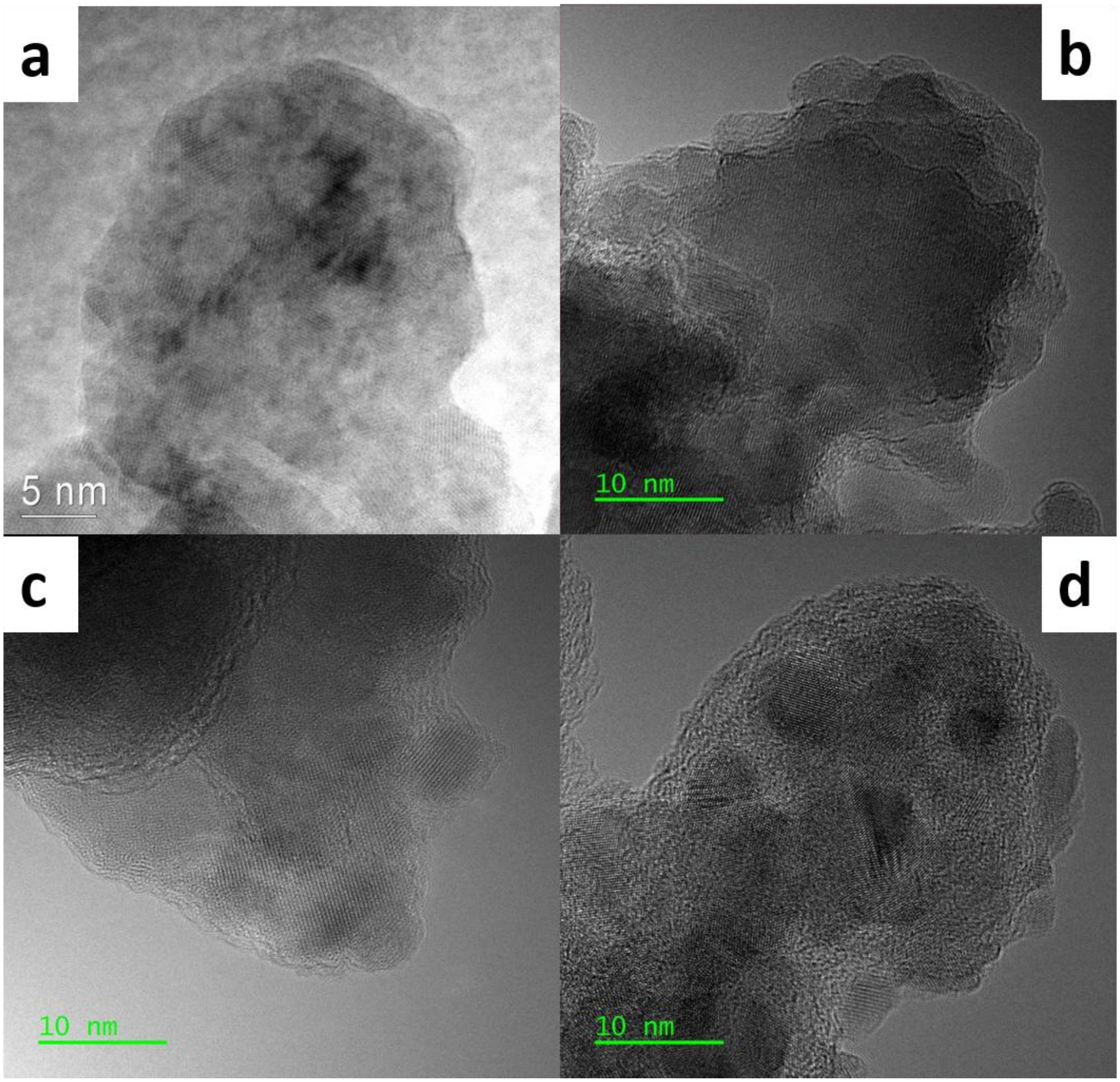

Figure S14: TEM of Cu_2O synthesized by bipolar electrochemistry for 1 hour at 4.5 V (a), 6.0 V (b), 7.0 V (c) and 8.0 V (d).

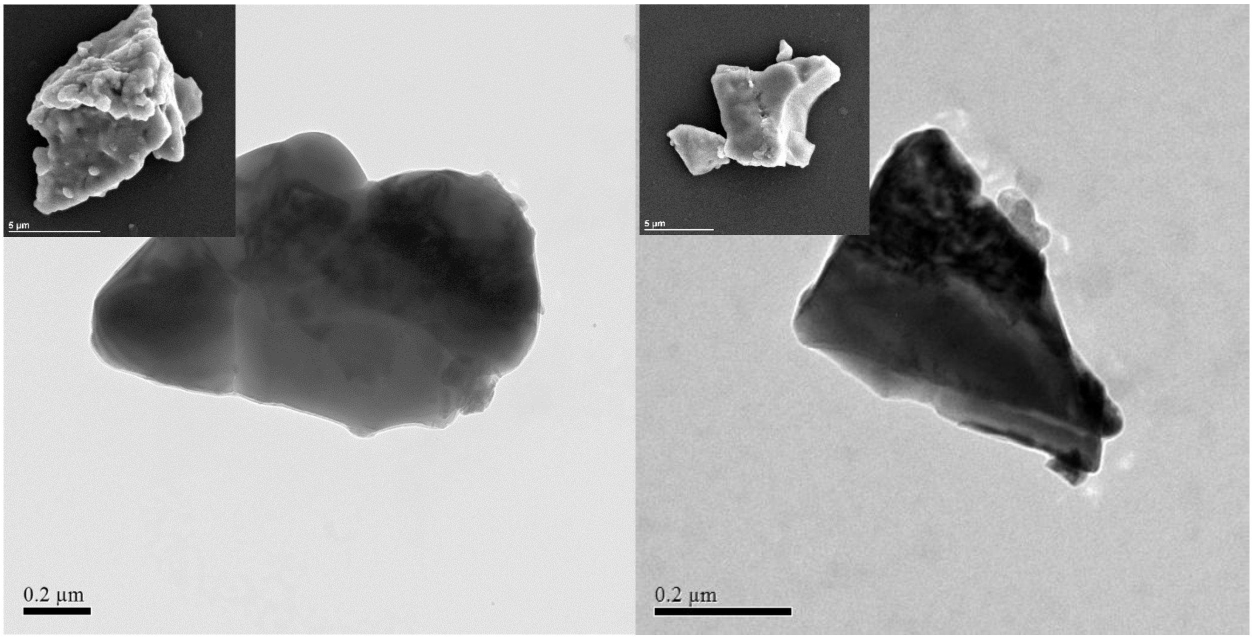

Figure S15: TEM and SEM (inserts) of commercial Cu_2O.

References

S1. M. L. Foo, Q. Huang, J. W. Lynn, W. -. Lee, T. Klimczuk, I. S. Hagemann, N. P. Ong and R. J. Cava, J. Solid State Chem., 2006, 179, 563.
